# Factors associated with low adherence to postpartum consultation: a cross-sectional study

**DOI:** 10.15649/cuidarte.4406

**Published:** 2025-05-01

**Authors:** Ana Luísa Macedo de Amorim, Chalana Duarte de Sena Fraga, Tacila Nogueira Azevedo Rocha, Kellen Karoline Almeida dos Santos Lira, Magna Santos Andrade

**Affiliations:** 1 Universidade do Estado da Bahia, Senhor do Bonfim, Brasil. E-mail: a.luisa.amorim8@gmail.com Universidade do Estado da Bahia Senhor do Bonfim Brasil a.luisa.amorim8@gmail.com; 2 Universidade do Estado da Bahia, Senhor do Bonfim, Brasil. E-mail: cdsena@uneb.br Universidade do Estado da Bahia Senhor do Bonfim Brasil cdsena@uneb.br; 3 Universidade do Estado da Bahia, Salvador, Brasil. E-mail: tacilanogueira@yahoo.com.br Universidade do Estado da Bahia Salvador Brasil tacilanogueira@yahoo.com.br; 4 Universidade do Estado da Bahia, Senhor do Bonfim, Brasil. E-mail: kellenkaroline2011@hotmail.com Universidade do Estado da Bahia Senhor do Bonfim Brasil kellenkaroline2011@hotmail.com; 5 Universidade do Estado da Bahia, Senhor do Bonfim, Brasil. E-mail: msandrade@uneb.br Universidade do Estado da Bahia Senhor do Bonfim Brasil msandrade@uneb.br

**Keywords:** Comprehensive Health Care, Primary Health Care, Postpartum Period, Women's Health, Atención Integral de Salud, Atención Primaria de Salud, Periodo Posparto, Salud de la Mujer, Assistência Integral à Saúde, Atenção Primária à Saúde, Período Pós-Parto, Saúde da Mulher

## Abstract

**Introduction:**

One of the attributes that attest to the effectiveness of postpartum care is the longitudinality of health care, which must be offered by Primary Health Care, an entity responsible for offering support and attention to maternal demands or women's health conditions.

**Objective:**

To investigate the frequency of postpartum consultations and the factors associated with low adherence to follow-up among postpartum women living in a municipality in the interior of the Northeast.

**Materials and Methods:**

Cross-sectional study, developed in the urban area of the municipality of Senhor do Bonfim, Bahia, Brazil, between June 2019 and January 2020. 97 women were surveyed, based on semi-structured interviews. The Chi-square/Fisher's Exact and Multiple Logistic Regression tests were used to perform data analysis.

**Results:**

There was 67% attendance at the postpartum consultation. Furthermore, an association was found between not having attended a postpartum consultation and the following variables: having attended prenatal care at the Basic Health Unit (OR: 0.08; p=0.002) and not having received guidance during prenatal care about the importance of returning for postpartum follow-up (OR: 0.22; p=0.004).

**Discussion:**

It is important to highlight that even with the existence of national protocols, states and municipalities can implement measures to improve postpartum care based on their respective realities.

**Conclusion:**

A low frequency of postpartum consultations was observed among the women surveyed, and the main reason for non-attendance was the difficulty in going to the consultation due to lack of time. In addition, the lack of continuity of care after childbirth among women who attended prenatal care at the SUS stands out.

## Resumen

**Introducción:**

Uno de los atributos que certifica la efectividad de la atención puerperal es la longitudinalidad de la atención en salud, la cual debe ser ofrecida por la Atención Primaria de Salud, entidad encargada de ofrecer apoyo y atención a las demandas maternas o condiciones de salud de la mujer.

**Objetivo:**

Investigar la frecuencia de consultas posparto y los factores asociados a la baja adherencia al seguimiento entre puérperas residentes en una ciudad del interior del Nordeste brasileño.

**Materiales y métodos:**

Estudio transversal, desarrollado en el área urbana del municipio de SSenhor do Bonfim, Bahía, Brasil, entre junio de 2019 y enero de 2020. Se encuestaron 97 mujeres, mediante entrevistas semiestructuradas. Se utilizaron pruebas de Chi-cuadrado/regresión exacta de Fisher y regresión logística múltiple para realizar el análisis de datos.

**Resultados:**

Hubo 67% de asistencia a la consulta postparto. Además, se encontró asociación entre no haber asistido a consulta posparto y las siguientes variables: haber asistido a la atención prenatal en la Unidad Básica de Salud (OR: 0,08; p=0,002) y no haber recibido orientación durante la atención prenatal sobre la importancia de retornar para la atención posparto (OR: 0,22; p=0,004).

**Discusión:**

Es importante resaltar que, si bien existen protocolos nacionales, los estados y municipios pueden implementar medidas que mejoren la atención posparto de acuerdo con sus respectivas realidades.

**Conclusiones:**

Se observó una baja frecuencia de consultas postparto entre las mujeres encuestadas, siendo el principal motivo de inasistencia la dificultad para acudir a la consulta por falta de tiempo. Además, se destaca la falta de continuidad de la atención después del parto entre las mujeres que recibieron atención prenatal a través del SUS.

## Resumo

**Introdução:**

Um dos atributos que atestam a eficácia da assistência puerperal é a longitudinalidade do cuidado em saúde, que deve ser ofertada pela Atenção Primária a Saúde, entidade responsável por ofertar acolhimento e atenção às demandas maternas ou condições de saúde da mulher.

**Objetivo:**

Investigar a frequência da consulta de pós-parto e os fatores associados à baixa adesão do acompanhamento entre puérperas residentes de um município do interior do Nordeste.

**Materiais e métodos:**

Estudo transversal, desenvolvido na zona urbana do município de Senhor do Bonfim, Bahia, Brasil, entre junho de 2019 a janeiro de 2020. Foram pesquisadas 97 mulheres, a partir da entrevista semiestruturada. Os testes Qui- quadrado/Exato de Fisher e Regressão Logística Múltipla foram utilizados para realizar a análise de dados.

**Resultados:**

Houve 67% de comparecimento à consulta puerperal. Além disso, constatou-se associação entre não ter realizado consulta de pós-parto e as seguintes variáveis: ter realizado o acompanhamento pré- natal na Unidade Básica de Saúde (OR: 0,08; p=0,002) e não ter recebido orientação no pré-natal sobre a importância do retorno para o acompanhamento no pós-parto (OR: 0,22; p=0,004).

**Discussão:**

É importante destacar que mesmo existindo protocolos nacionais, os estados e municípios podem implementar medidas que melhorem a atenção no pós-parto a partir das respectivas realidades.

**Conclusões:**

Observou-se uma baixa frequência de consultas puerperais entre as mulheres pesquisadas, sendo que o principal motivo do não comparecimento foi a dificuldade de ir à consulta em virtude da falta de tempo. Além disso, destaca- se a falta da continuidade da assistência após o parto entre as mulheres que realizaram o pré-natal no SUS.

## Introduction

The puerperium, period experienced for the woman since the expulsion from the placenta up to an average of six weeks after giving birth, this is a delicate moment in a woman's life due to the physical, psychological and social changes experienced. In this scenario, the injuries, pain and trauma resulting from childbirth can debilitate in the absence of adequate care^1^-^2^. 

Changes that occur during the puerperal period may have metabolic, hormonal and emotional origins, which increases women's susceptibility to certain endogenous or exogenous problems. These complications include postpartum infection, postpartum depression, venous complications, breast and urinary infections, dyspareunia, puerperal hemorrhage, among others^3^-^4^. 

Female genital tract infection, for example, is a type of postpartum condition that can spread systemically and compromise the functioning of the body, resulting in the death of the woman within a short period of time. Timely identification and treatment of complications such as this are essential to prevent seriousness and maternal death^5^-^6^. 

In 2019, the Maternal Mortality Ratio (MMR) was 57.9 per 100,000 live births in Brazil, of which 20% were due to hypertension, 12,4% to hemorrhage and 4,4% to puerperal infection. Almost all deaths were due to preventable causes, since the ways to prevent and control these diseases are already well understood and can be accessed through quality health services^5^-^8^. 

One of the attributes for the effectiveness of postpartum care is the longitudinality of health care, one of the attributes of Primary Health Care (PHC). PHC corresponds to the set of actions, at an individual and collective level, that promote the recognition of the population's needs and the resolution of approximately 80% of the health problems of the population assisted^9^-^11^. 

Postpartum care involves welcoming and addressing maternal needs, with a focus on preventing, promoting, protecting and treating women's health problems or conditions. To this end, the nurse and/or physician must perform at least two postpartum consultations within forty-two days after delivery, in order to assess maternal health, identify complications and provide guidance on reproductive planning, breastfeeding and newborn care^5^,^9^. However, despite what is expected, the frequency of postpartum consultations is significantly low in Brazil. In Botucatu, São Paulo, for example, the proportion of women who attend postpartum consultations is 58.3%. In Uberaba, Minas Gerais, this figure corresponds to 34.7%^12^,^13^. 

In this context, research to identify the frequency and factors that hinder access to postpartum care is relevant for different Brazilian locations, as it can contribute to understanding different realities. Such knowledge can support the development and implementation of strategies to improve postpartum care for women. 

This study aims to objective to investigate the frequency of postpartum consultations, and the factors associated with low adherence to follow-up among postpartum women living in a municipality in the interior of the Northeast. 

## Materials and Methods

This is a cross-sectional, quantitative, descriptive-analytical study. The research was carried out in Senhor do Bonfim, Bahia, Brazil, a municipality with an estimated population of 79,424 inhabitants in 2020 and a Municipal Human Development Index (MHDI) of 0,666, which corresponds to a medium-level classificationv^14^.

Women were recruited for the study based on records from the Live Birth Information System (SINASC) of women who gave birth between March and June 2019 (the time frame adopted for this analysis), resulting in a total of 180 women. The initial proposal was that all 180 women who gave birth in the period described above would be surveyed. However, there were 83 losses, resulting in a final number of 97 participants. 

It is important to highlight that 54.30% (45) of these losses occurred due to inconsistencies found in SINASC (such as incomplete addresses), which made it impossible to locate them. As it is an important information system for the analysis of maternal and child health conditions, and since it is extremely important to correctly fill in information about live births, a second article was produced and published that discussed the quality of SINASC information based on the analysis of the data used in the present study^15^. 

Regarding the other losses, 27.70% (23) occurred due to a change of address (it was not possible to obtain the address of the new place of residence), 9.60% (8) of the puerperal women were not found at home (even after three attempts to visit each) and 8.40% (7) due to refusal to participate. 

The study included postpartum women living in the urban area of the municipality, who were monitored by both the public and private health services. The inclusion criteria adopted were: postpartum women aged 18 or over and who were between the third and sixth month postpartum. 

The exclusion criteria considered were: birth resulting in stillbirth; neonatal death (immediate or late); death of the child up to the time of the survey, as recent trauma could result in response bias. 

The form used to collect data was developed based on questions contained in a survey entitled “Prenatal Care on Your Cell Phone (PRENACEL)”, carried out with pregnant and postpartum women living in Ribeirão Preto – São Paulo^16^ and in a literature review on the subject. Before being used, the instrument was tested with ten women living in Senhor do Bonfim, who were postpartum and who were not part of the final study. Possible errors were then corrected, and the form was improved. 

Five undergraduate nursing students from the Universidade do Estado da Bahia (UNEB) who received prior training participated in data collection. Data collection took place from June 2019 to January 2020 through interviews conducted during home visits. 

Participants were informed about the objectives of the research, their voluntariness and the need for consent. Two copies of the Informed Consent Form were given and signed, one for the researcher and the other for the woman being interviewed. All participants who agreed to participate in the research participated in the study. The project was approved by the Research Ethics Committee (REC) in March 2019 under opinion number 3,212,217. The data and literature used for the theoretical basis of this study are freely available at Mendeley Data^17^. 

The data collected was then double entered into the Statistical Package for the Social Science (SPSS) software version 22 and checked by comparing the two banks of simple frequencies of all variables. Thus, inconsistencies related to typing could be corrected and those related to filling in the data were considered as losses. 

Initially, a descriptive analysis of the study was performed, based on the absolute and relative frequencies of the variables studied. Regarding the comparison analysis between groups, the outcome variable adopted was the completion of the “postpartum consultation”, categorized as having or not having attended the consultation. The independent variables were: sociodemographic, obstetric history and characteristics of prenatal care and current delivery. 

For the bivariate analysis, the following were calculated: Odds Ratio (OR), 95% Confidence Interval (CI) and Pearson's Chi -square/Fisher's Exact Test, with an association considered when the p-value is less than 0.05. 

Finally, an adjusted analysis was performed using Multiple Logistic Regression, with three types of models being calculated to verify which one best fit the proposed analysis: Model 1 - all independent variables were used for the analysis; Model 2 - Stepwise ; Model 3 - only those independent variables whose p-value of the Chi -square/Fisher's Exact test was less than 0.25 were included in the analysis. The best-fitting model for this analysis was Stepwise, but it is important to highlight that all three models found an association between the outcome variable and the same independent variables, which further reinforces the results obtained. 

It is reiterated that this research was financed by the Bahia State Research Support Foundation (FAPESB), associated with the larger project entitled “Postpartum reproductive planning among women treated in Primary Care (PC) in Senhor do Bonfim – BA”^18^. 

Strengthening checklist was used. the Reporting of Observational Studies in Epidemiology^19^. 

## Results

Regarding the sociodemographic characteristics of the 97 women surveyed, 75.20% were under 35 years of age, 80.40% self-identified as black or brown, 88.50% had eight or more years of schooling, 82.50% belonged to social classes C, D or E and 50.50% performed some type of paid activity. No associations were observed between the variables analyzed (Table 1). 

**Table 1:** Sociodemographic characteristics of postpartum women in the municipality of Senhor do Bonfim-BA, 2019-2020

Sociodemographic variables	Total n(%)	Postpartum consultation		OR (IC 95%)	Value p1
		Yes n(%)	No n(%)		
Age (years)					
< 35	73 (75.20)	46 (70.80)	27 (84.40)	2.23 (0.75 – 6.66)	0.144
≥ 35	24 (24.80)	19 (29.20)	05 (15.60)	1	
Race/color					
Black/brown	78 (80.40)	50 (76.90)	28 (87.50)	2.10 (0.63 – 6.94)	0.282
White/yellow	19 (19.60)	15 (23.10)	04 (12.50)	1	
Number of children					
1-2	76 (78.40)	49 (75.40)	27 (84.40)	1.76 (0.58 – 5.34)	0.316
≥ 3	21 (21.60)	16 (24.60)	05 (15.60)	1	
Education					
≤ 7	11 (11.50)	07 (10.90)	04 (12.50)	1.16 (0.31 – 4.30)	1
≥ 8	85 (88.50)	57 (89.10)	28 (87.50)	1	
Class Social 2					
A and B	17 (17.50)	13 (20)	04 (12.50)	1	
C, D and E	80 (82.50)	52 (80)	28 (87.50)	1.75 (0.52 – 5.87)	0.411
State civil					
With partner	83 (85.60)	58 (89.20)	25 (78.10)	1	
Without partner	14 (14.40)	07 (10.80)	07 (21.90)	2.32 (0.73 – 7.31)	0.143
He has religion					
Yes	72 (75.00)	52 (81.20)	20 (62.50)	1	
No	24 (25.00)	12 (18.80)	12 (37.50)	2.6 (1 – 6.74)	0.045
Which religion					
Catholic	49 (68.10)	36 (69.20)	13 (65.00)	0.82 (0.27 – 2.45)	0.730
Protestant	23 (31.90)	16 (30.80)	07 (35.00)	1	
Head of the family					
Partner	58 (77.30)	44 (80)	14 (70)	1	
Own woman	17 (22.70)	11 (20)	06 (30)	1.71 (0.53 – 5.48)	0.360
Has a paid job					
Yes	49 (50.50)	36 (55.40)	13 (40.60)	0.55 (0.23 – 1.30)	0.171
No	48 (49.50)	29 (44.60)	19 (59.40)	1	
Activity current					
Autonomous	26 (53.10)	19 (52.80)	07 (53.80)	1	
Employee/salaried	23 (46.90)	17 (47.20)	06 (47.20)	0.95 (0.26 – 3.41)	0.947

1 Value found using Pearson's Chi -square/Fisher's Exact Test. 2 Income monthly average of the classes A (> 22 wages minimum), B (≥ 5 wages ≤ 22 wages) C (≥ 2 Salaries ≤ 5 wages) D and E (< 1 wage). The minimum wage in Brazil was R$998,00 in 2019.

Furthermore, about the puerperal consultation, 67.00% attended the postpartum consultation, and 73.80% attended only one appointment. Among the main reasons that justified non-adherence, 25.00% of puerperal women reported the lack of time to schedule the appointment, 18.80% mentioned the lack of time to go to the consultation and 15.60% stated a lack of interest in attending the unit (Table 2). 

According to the information on the pregnancy-puerperal period, 82.30% of participants had one to two previous pregnancies, 77.90% had seven or more prenatal appointment, of which 72.90% were in the Sistema Único de Saúde (SUS), 55.70% had a cesarean section and 60.80% received guidance on the importance of returning for the puerperal consultation during prenatal care. Regarding the association between the variables, it was observed that the chance of not having a puerperal consultation among women who did not schedule the consultation at the maternity hospital was 5.47 times higher compared to women who did schedule it (Table 3). 

Regarding guidance during prenatal care about postpartum appointment, the chance of not attending postpartum follow-up is 2.89 times greater among those who did not receive the information compared to the chance among women who were guided during pregnancy. When analyzing the association between not attending postpartum consultations and the type of service where the woman received prenatal care, the chance of not returning for postpartum follow-up among puerperal women who received prenatal care through the SUS is 5.42 times greater than the chance among those who received care through private services (Table 3). 

**Table 2:** Aspects of postpartum consultation among postpartum women in the municipality of Senhor do Bonfim-BA, 2019-2020

Postpartum consultation	Postpartum women	
	n	%
She went to the appointment (n=97)		
Yes	65	67.00
No	32	33.00
Number of appointments made (n=65)		
One appointment	48	73.80
Two appointments	17	26.20
Reasons for not joining (n=32)		
Lack of time to make an appointment	08	25.00
Lack of time to go to the appointment	06	18.80
Not interested in going to the appointment	05	15.60
She didn't know I had to go	03	9.40
She didn't know when to go	01	3.10
UBS does not offer follow-up	01	3.10
Indisposition	01	3.10
Others factors	07	21.90

**Table 3:** Obstetric history, prenatal characteristics and current delivery of postpartum women in the municipality of Senhor do Bonfim-BA, 2019-2020

Variables of pregnancy and childbirth	Total n(%)	Postpartum Consultation		OR (95% CI)	p-value1
		Yes n(%)	No n(%)		
Pregnancies previous					
1-2	56 (82.30)	35 (79.50)	21 (87.50)	1	
≥ 3	12 (17.70)	09 (20.50)	03 (12.50)	0.55 (0.13 – 2.28)	0.517
Current pregnancy planned					
Yes	51 (52.60)	36 (55.40)	15 (46.90)	1	
No	46 (47.40)	29 (44.60)	17 (53.10)	1.40 (0.60 – 3.28)	0.430
Prenatal in gestation current					
Yes	96 (99.00)	64 (98.50)	32 (100)	*	*
No	01 (1.00)	01 (1.50)	0		
Prenatal appointment					
≤ 6	21 (22.10)	15 (23.80)	06 (18.80)	0.73 (0.25 – 2.13)	0.663
≥ 7	74 (77.90)	48 (76.20)	26 (81.20)	1	
Scheduling of the postpartum appointment at the maternity hospital					
Yes	20 (21.30)	18 (28.10)	02 (6.70)	1	
No	74 (78.70)	46 (71.90)	28 (93.30)	5.47 (1.18 – 25.41)	0.028
Guidance in the PN on the postpartum appointment					
Yes	59 (60.80)	45 (69.20)	14 (43.80)	1	
No	38 (39.20)	20 (30.80)	18 (56.20)	2.89 (1.20 – 6.93)	0.015
Location of prenatal					
SUS**	70 (72.90)	41 (64.10)	29 (90.60)	5.42 (1.48 – 19.77)	0.006
Private Service	26 (27.10)	23 (35.90)	03 (9.40)	1	
Current birth route					
Vaginal	43 (44.30)	25 (38.50)	18 (56.30)	2.05 (0.87 – 4.85)	0.097
Caesarean section	54 (55.70)	40 (61.50)	14 (43.70)	1	

1 Value found using Pearson's Chi -square/Fisher's Exact Test. CI – Confidence Interval; OR – Odds Ratio; SUS – Brazilian National Health System; PN – Prenatal; * No OR or CI was calculated because there was only one person surveyed in one of the categories; **Consultation carried out in the Family Health Strategy or other public services.

 The chance of not returning for the postpartum appointment among women who received prenatal care through the SUS is 0.05 in relation to the chance of those who received follow-up care in the private service. Among the puerperal women who did not receive guidance during prenatal care about postpartum follow-up, the chance of not attending the consultation is 0.20 in relation to those who received guidance (Table 4).

**Table 4:** Adjusted analysis of the variable’s location of the PN and guidance on the puerperal consultation in the municipality of Senhor do Bonfim-BA, 2019-2020

Variables	Model with all variables1		Stepwise Model2		Model variables p < 0.253	
	OR (95% CI)	p	OR (95% CI)	p	OR (95% CI)	p
PN location						
SUS	0.05 (0.01-0.41)	0.007	0.08 (0.01-0.41)	0.002	0.12 (0.03-0.50)	0.004
Private	1		1		1	
Guidance on postpartum appointment						
Yes	1		1		1	
No	0.20 (0.06-0.62)	0.005	0.22 (0.08-0.63)	0.004	0.23 (0.08-0.64)	0.005

1 All independent variables were used for the analysis. 2 Stepwise. 3 Only those independent variables whose Chi -square/ Fisher's exact p-value was less than 0.25 were included in the analysis. PN – Prenatal.

## Discussion

In this study, we investigated the factors associated with low adherence to postpartum appointment among women in the interior of the Northeast, also listing the frequency of these consultations. The results show that prenatal care was carried out in a public network, the lack of guidance on the importance of returning for the postpartum appointment and the failure to schedule the consultation by the maternity hospital are factors that stand out as promoters of non-attendance at postpartum follow-up. 

In this sense, regarding postpartum monitoring, Baratieri and Natal (2019) state that, according to the indicators of postpartum appointment, Brazil stands out as having low performance, with percentages ranging from 16.80% to 58.00%. However, Peru (in its 9 poorest regions) also had 58.00% of women who underwent postpartum monitoring, while in the United Kingdom the percentage reached 91.00% in six weeks postpartum. In Australia, the impasses referred to the lack of adherence to postpartum appointment were related to the lack of guidelines and protocols for care for the mother-child binomial^20^. 

In the Northeast, a study indicates that the access variables that most influence adherence to postpartum appointment are the distance from the residence to UBS and information about the UBS's opening hours. A higher prevalence of consultations was identified for women who lived less than 16 minutes from the UBS. The fact that the woman knew the opening hours and the daily availability on five days a week was also able to increase the chance of attending the consultation by 1.30^21^. 

The same study showed that sociodemographic variables also act as determinants, since women living in the South and Southeast were more likely to have a postpartum appointment, as were those with a high school diploma^21^. 

Among the women interviewed in Senhor do Bonfim, 67.00% attended at least one postpartum follow-up appointment, a value similar to that found for the Northeast region, according to the National Base Study Nascer no Brasil (62.80%)^22^. 

The aforementioned study also found that the frequency of return for at least one postpartum appointment in the country was 73.90%, and could vary from 57.20% (states that make up the North region) to 87.00% (South region)^22^. 

In Brazil, there is no isolated indicator of minimum coverage recommended to be achieved in relation to the frequency of postpartum appointment according to the target audience, which shows the lack of prioritization by the state in relation to the monitoring of postpartum women. 

Among the women interviewed in Senhor do Bonfim, 67.00% received health care after childbirth, a value similar to that found for the Northeast region, according to the study mentioned above (62.80%)^22^. 

It is important to highlight that even though there are national protocols, states and municipalities can implement measures that improve postpartum care based on their respective realities, as is the case in Rio Grande do Sul with resolution 251 of 2018. This regulation includes, as one of the measures to guarantee monitoring during the pregnancy-puerperal cycle, at least one postpartum consultation up to the twentieth day postpartum^23^. 

Another important result of this research was the fact that less than a third of the puerperal women who attended the first consultation returned for a second appointment. When searching for data regarding this return in other contexts, a lack of such information was observed, which reinforces the need to devote greater attention to postpartum care. 

The HM recommends carrying out two postpartum consultations, one of which should be carried out in the first week after birth, preferably in the form of a home visit, and the other within forty- two days after childbirth^1^,^24^. Furthermore, it reinforces the need to identify the reasons of non- attendance, of actively searching for the absent puerperal woman and of offering a new day to the query^5^. 

In this context, Community Health Agents (CHAs) play a fundamental role, being able to provide guidance on the importance of consultations, encourage return visits and strengthen the link between the health service and the woman^25^. 

In addition, the health team can define strategies to increase adherence among postpartum women, such as providing alternative times for care at the unit and providing information about the possibility of taking the newborn to the outpatient clinic at the time of the consultation. 

When mentioning the reasons that led to the non-performance of postpartum monitoring, almost half of the participants in the municipality surveyed mentioned issues related to lack of time. A qualitative study on the experiences of women in the postpartum period, carried out in the northwest of Paraná, pointed to the lack of time as one of the main difficulties encountered in the postpartum period, which can be justified by aspects such as the reduced support network and the social stereotype of the devoted maternal figure, of sacrifice, of donation and of exclusive dedication to the newborn^26^. 

In a study carried out in the city of Uberaba, Minas Gerais, the main reasons given by postpartum women for not having attended the postpartum appointment were forgetfulness and the emergence of complications with the newborn^13^. 

In the present analysis, an association was observed between prenatal care being carried out in the SUS and the puerperal woman not returning for the postpartum appointment. This result may be related to some aspects mentioned in the literature, such as the breakdown of the bond between the primary care professional and the user (due to the high turnover of health team members), the difficulty in obtaining care in the SUS and the long distance between the health unit and homes^27^,^28^. 

Furthermore, it was noted that in the municipality studied there was a relationship between women not having been informed about the importance of postpartum monitoring during prenatal care and not having attended a postpartum appointment. A study carried out in 2019 showed the inadequacy of the guidance received by women during prenatal care as one of the problems found in primary care in the state of Santa Catarina^29^. 

Health education is extremely important in all aspects of primary care, as it contributes to the prevention and reduction of injuries, diseases and complications. Scenarios in which the transfer of information in maternal care is unsatisfactory can result in a worsening of quality of life levels during pregnancy, childbirth and the postpartum period, and consequently in the risk to the maintenance of life^30^. 

Maternal health care, therefore, should be seen as a line of care that goes from pre-conception monitoring to the postpartum period^31^. To this end, the professionals involved must promote longitudinal and uninterrupted care throughout all phases of the pregnancy-puerperal period. 

The advantage of this study is that it analyzes in detail aspects related to the coverage of puerperal monitoring in the municipality's Primary Care, as these aspects are not included in the health information systems and are valuable information for local management in the search for improving the quality of maternal health care. 

One limitation observed is the high percentage of losses due to data related to the identification of postpartum women in SINASC, which led to a significant reduction in the group to be researched. 

## Conclusions

A low frequency of postpartum appointment was observed among the women surveyed, with the main reason for non-attendance being the difficulty in going to the consultation due to lack of time. In addition, the lack of continuity of care after childbirth among women who received prenatal care in the SUS stands out, even though the primary care has an essential tool, the CHAs, who goes door to door and is the main actor in the active search for those who are absent. 

Another important point was the limited use of health education to raise awareness among women about returning to the health service after giving birth, a worrying finding, as both individual and group education should be one of the foundations of the care offered by Family Health Teams in serving the community. 

Failure to attend postpartum appointments has significant impacts on the health of the mother- child pair. In addition to compromising the monitoring of conditions identified during pregnancy, which may worsen or become chronic, it also interferes with adherence to reproductive planning and childcare consultations and compliance with the child's vaccination schedule. Studies such as this one, which identify these gaps, are essential for the development of strategies that promote the bond between the patient and the primary care health team. These strategies include educational measures on the importance of continuity of care in the postpartum period, encouraging active search and recruitment of women who are absent, as well as training professionals responsible for increasing adherence to postpartum appointment, contributing, in the long term, to improving public health. 

Thus, it is expected that the gaps highlighted by this study will serve as a basis for formulating strategies that increase the coverage and quality of postpartum appointment, with the aim of maintaining longitudinal care even after childbirth. 
